# Biochemical characterization and insights into the potency of the acidic *Aspergillus niger* NRC114 purified α-galactosidase in removing raffinose family oligosaccharides from soymilk yogurt

**DOI:** 10.1186/s12896-023-00773-x

**Published:** 2023-01-31

**Authors:** Abdelmageed M. Othman, Ali M. Elshafei, Maysa A. Elsayed, Gamil E. Ibrahim, Mohamed M. Hassan, Nayra S. Mehanna

**Affiliations:** 1 Microbial Chemistry Department, Biotechnology Research Institute, National Research Centre, 33 El Bohouth St., Dokki 12622 Giza, Egypt; 2 Chemistry of Flavor and Aroma Department, Food Industries and Nutrition Research Institute, National Research Centre, 33 El Bohouth St., Dokki 12622 Giza, Egypt; 3 Dairy Sciences Department, Food Industries and Nutrition Research Institute, National Research Centre, 33 El Bohouth St., Dokki 12622 Giza, Egypt

**Keywords:** α-galactosidase, Purification, Soy bean, Nutritional properties, Treated-soymilk yogurt

## Abstract

**Background:**

Because humans lack α-galactosidase, foods containing certain oligosaccharides from the raffinose family, such as soybeans and other legumes, may disrupt digestion and cause flatulence.

**Results:**

*Aspergillus niger* NRC114 α-galactosidase was purified using protein precipitation, gel filtration, and ion exchange chromatography steps, which resulted in a 123-fold purification. The purified enzyme was found to be 64 kDa using the SDS-PAGE approach. The optimum pH and temperature of the purified α-galactosidase were detected at pH 3.5 and 60 ºC, respectively. The pure enzyme exhibited potent acidic pH stability at pH 3.0 and pH 4.0 for 2 h, and it retained its full activity at 50 ºC and 60 ºC for 120 min and 90 min, respectively. The enzyme was activated using 2.5 mM of K^+^, Mg^2+^, Co^2+^, or Zn^2+^ by 14%, 23%, 28%, and 11%, respectively. The *K*_*m*_ and *V*_*max*_ values of the purified enzyme were calculated to be 0.401 µM and 14.65 μmol min^−1^, respectively. The soymilk yogurt showed an increase in its total phenolic content and total flavonoids after enzyme treatment, as well as several volatile compounds that were detected and identified using GC–MS analysis. HPLC analysis clarified the enzymatic action in the hydrolysis of raffinose family oligosaccharides.

**Conclusion:**

The findings of this study indicate the importance of *A. niger* NRC114 α-galactosidase enzyme for future studies, especially its applications in a variety of biological fields.

**Supplementary Information:**

The online version contains supplementary material available at 10.1186/s12896-023-00773-x.

## Introduction

α-Galactosidase (α-D-galactoside galactohydrolase, EC 3.2.1.22) is a hydrolytic enzyme that catalyzes the degradation of terminal α-1,6-linked D-galactose residues in galactooligosaccharides and galactopolysaccharides [1]. It has miscellaneous usage in the nutraceutical, pharmaceutical industries and other industrial fields. α-Galactosidase has a great role in the sugar industry due to its catalytic degradation of raffinose sugar, which enhances the crystallization of sucrose and increases the product [2]. The enzyme is a good participant in soy food processing and animal feed processing [3]. At the level of medical applications, α-galactosidase was studied for the process of blood group transformation and the treatment of Fabry’s disease [4].

Although the α-galactosidase enzyme is excessively spread in most living systems, it is not formed in humans. In plants, α-Galactosidase has been studied in *Vigna mungo* [5] and *Citrullus vulgaris* [6]. It has been immobilized by the bacterium *Thermus sp.* T2 [7], is secreted by *Lactobacilli* during the fermentation of soy milk [8], and produced by lactic acid bacteria [9]. The purified enzyme from *Bacillus stearothermophilus* (NCIM-5146) could catalyze the hydrolysis of raffinose and stachyose [10].

Nowadays, many researchers and industrialists have given more attention to microbial enzymes in several industrial fields. These enzymes are characterized by low-cost production, plentiful yields, stability at a wide range of pH values and temperatures, and independence from seasonal production because the microbes have the ability to grow on inexpensive media and produce the enzymes under harsh circumstances [11]. Filamentous fungi and mushrooms are considered excellent sources for α-galactosidases production because they grow easily on different agriculture debris. α-galactosidases produced by fungi are characterized by their extracellular secretion and broad stability profiles [12].

It is well known that soybeans and other legumes provide humans with food rich in proteins and other valuable nutrients. Consequently, there is a growing demand for these meals of plant origin to minimize the bad effects of other foods from animal sources. Feeding on soybeans and other legumes as they contain some oligosaccharides of the raffinose family can disturb the digestion process and cause flatulence due to the absence of α-galactosidase in humans. Processing of legumes and their products by α-galactosidase can be used to degrade such oligosaccharides to monosaccharides which improve the quality and taste of these products. Substantial efforts and various studies have been made to eliminate or reduce the beany off-flavor, maximize the yield, and extend the soymilk shelf life [13].

Recently, there has been an increasing demand for functional foods due to their therapeutic and biological characteristics as well as their nutritional value. Among the preferred functional foods, yogurt is preferred because of the presence of lactic acid bacteria, which are responsible for the domestic therapeutic properties during the fermentation process of milk and provide significant digestible nutrients [14]. Soybean is one of the most important legumes that are considered functional foods, with significant consumption worldwide. It was characterized by a high content of protein, minerals, vitamins, and fiber. The healthy properties like antioxidant activity, lowering cholesterol, and reduction of heart diseases of soybeans and prepared products come from biological components such as isoflavones, saponins, phytosterols, and vitamin E. However, soybean and processed products' flavors are considered "off-flavor" with a negative effect on sensory properties. In the food industry, several attempts have been made to eliminate this undesirable off-flavor through enzyme treatments.

Purification procedures are created to obtain an enzyme in a timely, cost-effective, and pure manner. The present study aims to get the α-galactosidase enzyme in a pure form and study its properties to be a guide for its ability in the treatment of soymilk yogurt to improve its taste and increase its nutritive value. Accordingly, the changes in chemical composition, phytochemicals, antioxidant activity as well as volatile compounds of yogurt prepared from soybean after treatment with α-galactosidase and also the overall acceptability in comparison with plain yogurt was evaluated.

## Materials and methods

### Chemicals

Glucose, raffinose, sucrose, stachyose, galactose, *p*-nitrophenyl α-galactopyranoside (α-p-NPGal), and fructose were obtained from Sigma chemical company. Agar was provided by Fluka, Spain. All the other chemicals used were of analytical grade.

### α-galactosidase activity assay

The activity of α-galactosidase was assessed in a reaction mixture contained: 0.2 ml of 4 mM *p*-nitrophenyl-α-D-galactopyranoside (*p*NP-α-D-Gal), 0.2 ml of 0.2 M citrate phosphate buffer (pH 3.5), and 0.1 ml of enzyme solution. The reaction was stopped after 10 min at 50 °C by adding 2 ml of a 0.2 M Na_2_CO_3_ solution. The released *p*-nitrophenol (*p*NP) was measured using a UV–Visible spectrophotometer (Cary 100 UV–Vis; Agilent Technologies, Germany) at 420 nm [12, 15]. One unit of activity was defined as the amount of enzyme capable of releasing 1 µmole of PNP per min. The protein content was measured at 595 nm using the Bradford method [16] and bovine serum albumin as a standard protein.

### α-galactosidase purification

*Aspergillus niger* NRC114 was grown on media adjusted to the parameters referenced according to the application of central composite design (CCD) [12]. Cell-free filtrate (CFF) of the aforementioned culture was filtered by using Whatman filter paper No.1, which is considered as the crude α-galactosidase. The first step in the purification process was accomplished by adding ammonium sulfate (60–90% saturation) to precipitate the protein content in the filtrate. Using a HERMLE Z-323 K cooling centrifuge, the precipitation mixture was spun for 15 min at 12,000 rpm. The precipitate was re-dissolved in a 50 mM citrate phosphate buffer (pH 4.5) and dialyzed against distilled water overnight at 4 °C. After pre-equilibrating with the preceding buffer, the dialyzed enzyme was put onto a (2.5 × 50 cm) Sephadex G100 gel filtration chromatography column. Using a 50 mM citrate phosphate buffer (pH 4.5) at a flow rate of 1.5 ml/min, fractions containing α-galactosidase enzyme were collected using LKB Bromma 2070 Ultrorac® Fraction Collector. The α -galactosidase-active fractions were then combined, concentrated, and applied to a (1.5 × 35 cm) DEAE-Sephadex A-50 anion exchange chromatography column that had been pre-equilibrated with 50 mM citrate phosphate buffer (pH 4.5). The column was then eluted with the same buffer at a rate of 0.5 ml/min using a gradient of NaCl concentrations ranging from 0 to 1.0 M. The elution of resulted protein peaks were observed at A_280_ nm. The resulting fractions containing the active, purified α-galactosidase were collected and kept at − 20 °C for use in further studies.

### Gel electrophoresis

To assess the effectiveness of the purification processes and determine the molecular weight of the enzyme, sodium dodecyl sulphate polyacrylamide gel electrophoresis (SDS-PAGE) was used. According to Laemmli [17], SDS-PAGE was accomplished using 12% polyacrylamide. Using a 3 kDa MWCO Millipore Amicon® Ultra-15 centrifugal filter (Sigma-Aldrich, USA), protein sample concentration was achieved. It was carried out using the EZ-RunTM Pre-stained Rec Protein Ladder (Fisher, Bioreagents TM; BP3603). After 3 h of Coomassie Brilliant Blue-R250 staining, the gel was destained using a 40:10% solution of acetic acid and ethanol, respectively.

### Temperature and pH dependency and stability of α-galactosidase

The purified enzyme (25.39 U/mL) was added to the substrate α-*p*-NPGal with the appropriate buffer and subjected to temperature values between 20 and 90 °C to detect the optimum temperature for the enzyme activity. The thermal stability of the enzyme was tested at 50–70 °C in the absence of the substrate over a time range of 10–120 min in a citrate phosphate buffer (50 mM, pH 3.5). The optimum pH value for the enzyme activity was determined by using Robinson buffer at pH values in the range of 2.0–7.0. The pH stability was revealed by incubating the enzyme with the same buffer at pH values of 3.0–7.0 at two different temperatures (30 °C and 60 °C) and different time intervals (15–120 min).

### Effect of various metal ions on α-galactosidase activity

The effects of various metal ions, namely: MnCl_2,_ HgCl_2_, CaCl_2,_ KCl, MgSO_4_, CoSO_4_, ZnSO_4_, CuSO_4_, and FeSO_4_, on the activity of the pure enzyme (25.39 U/mL), were determined. EDTA was added to the reaction mixture to determine whether it required a metal ion to complete the catalytic process of the enzyme or not. The α-galactosidase activity in the absence of metal ions was recorded as 100%.

### Kinetic studies

Using *p*NP-α-D-Gal as a substrate, the kinetic constants (*K*_*m*_ and *V*_*max*_) for α-galactosidase were calculated at pH 3.5 and 60 °C. The Michaelis–Menten constant (*K*_*m*_) was identified to express the affinity of α-galactosidase for its substrate. The *V*_*max*_ value was calculated by incubating the same amount of the enzyme (25.39 U/mL) and varying the substrate concentrations in the range of (0.04–0.8 µM) at the initial rate of hydrolysis of the substrate. The tests were performed in triplicate. The Lineweaver–Burk plots were created using the GraphPad Prism program to determine the *K*_*m*_ and *V*_*max*_ values.

### Soymilk yogurt drink preparation

Soymilk was prepared as described by Elshafei et al. [12] and pasteurized at 72 °C for 15 s., then cooled down in an ice bath, and then inoculated with 10% of an activated probiotic mixture including *Lactobacillus acidophilus* CUL60, *Bifidobacterium lactis* HNO19, and *Bifidobacterium bifidum* CUL20. The inoculated soymilk was incubated at 42 °C for 24 h in sterilized bottles. After incubation for 24 h, the yogurt drink was stored in a refrigerator for 2 weeks.

### Treatment of soymilk yogurt by the pure α-galactosidase

Ten milliliters of the pure α-galactosidase enzyme (25.39 U/mL) was added to 60 ml of soymilk yogurt in an Erlenmeyer flask 250 ml. This mixture was incubated for 8 h at 50 °C in a shaking state (200 rpm). At the end of the incubation period, the enzymatic activity was suppressed in a boiling water bath for 10 min.

### Assessment of the proximate chemical composition of yogurt samples

Using the recommended techniques of the Association of Official Analytical Chemists [18], the proximate compositions of experimental soy yogurt and plain yogurt were measured in triplicate. The Bligh and Dyer [19] technique was used to calculate fat. Carbohydrate content was calculated according to the equation:

$${\text{Utilized Carbohydrate }}\% \, = { 1}00 \, - \, \% \, \left( {{\text{moisture }} + {\text{ crude protein }} + {\text{ lipid }} + {\text{ ash}}} \right).$$

### Determination of pH and titratable acidity

Yogurt samples, weighing 10 g each, were diluted in 10 mL of deionized water to detect the pH value using a pH meter (Hanna pH-meter HI 9021, Germany). These samples were subjected to a titration process using 0.01 N NaOH solution and 5 drops of 5 g/100 mL phenolphthalein solution. The total titratable acidity (TTA) was then calculated as lactic acid equivalents (g/L) as follows:

$${\text{TTA }} = {\text{ Dilution factor }}\left( {{1}0} \right) \, \times {\text{ V}}_{{({\text{NaOH}})}} \times \, 0.0{\text{1 N }} \times \, 0.00{9 } \times { 1}00$$

### Phytochemicals and antioxidant activity

#### Preparation of yogurt water extract

Yogurt was combined with distilled water in a 1: 0.25 ratio to create yogurt water extract, which was then centrifuged (6708 xg, for 10 min) at 4 °C after being acidified to a pH of 4.0 with 1 M HCl and incubated at 45 °C for 10 min in a water bath. Thereafter, the supernatant was adjusted to pH 7.0 using 0.5 M of NaOH. Within 12 h of preparation, a further stage of centrifugation was performed to obtain yogurt water extract for use in pertinent experiments [20]**.**

### Total phenolic assay

Shetty et al. [21] methodology was used to calculate the total phenolic content (TPC). In which diluted Folin-Ciocalteu reagent was added to yogurt water extracts and a 95% ethanol mixture. A Shimadzu UV 1601 spectrophotometer (Japan) was used to determine the absorbance at 725 nm. Gallic acid was employed as a standard to represent the data as total phenolics, where gallic acid equivalents in micrograms per gram (µg GAE g^−1^) of sample were used.

### Determination of total flavonoids

The total flavonoid content of yogurt samples was determined according to Barros et al. [22]. Total flavonoid content was calculated on the basis of the rutin calibration curve and represented as microgram equivalents of rutin per gram (µg RE g^−1^) of the sample [12].

### Antioxidant activity using ABTS radical scavenging and ferric-reducing power

To evaluate the yogurt samples' ability to scavenge the ABTS radical in a reaction, Re et al. [23] technique was used. In which, 2, 2'-Azino-bis (3-ethylbenzothiazoline-6-sulfonic acid) diammonium salt stock solution was oxidized with potassium persulfate (K_2_S_2_O_8_) to produce the ABTS radical solution. On the other hand, the protocol outlined by Benzie and Strain [24] was followed to conduct the ferric-reducing antioxidant power (FRAP) test. Ascorbic acid was used to create the calibration curve, and the findings were given as micrograms of ascorbic acid equivalents (AAE) per milliliter of extract.

### Sugar analysis using HPLC

Absolute ethanol was added to large conical centrifuge tubes containing 10 g of yogurt samples to achieve a final ethanol concentration of 80% (v/v). To precipitate the proteins, slurries were combined and let stand for 20 min at room temperature. A total of 50 ml of ethanol (80% v/v) was then added. The supernatant was obtained after centrifuging the samples at 5000 xg for 5 min. The precipitate was washed with a further 25 mL of an 80% ethanol solution. Following that, the supernatants were all concentrated using a rotary vacuum evaporator (25 to 27 °C). In order to prepare the samples for eventual HPLC analysis, they were filtered using a 0.45 µm Metricel membrane, put in vials, sealed, and frozen at -10 °C. A Shimadzu HPLC Class-VPV 5.03 (Kyoto, Japan) was used to analyze the sugar in the filtrate. HPLC specifications and work conditions were as mentioned in Elshafei et al. [12].

### Volatile compounds analysis by GC–MS

Yogurt samples (10 g) were mixed with NaCl (16% w/v) for mild shearing, then transferred to a volumetric flask, fixed to 100 mL with NaCl solution, and well shaken. The Solid Phase Microextraction (SPME) fiber was placed in a 20-mL headspace vial with processed samples (5 mL), which were then immediately sealed. The headspace vial was extracted for 30 min while being stirred at 200 rpm after being equilibrated in a water bath at a constant temperature for 15 min at 60 °C. An Agilent 7890 GC connected to a 5977 MS detector was used for the analyses. The GC–MS operating conditions and compounds recognition were achieved as described by Elshafei et al. [12].

### Sensory assessment

As specified by Yang and Li [25], the plain and soy yogurt samples underwent sensory and an organoleptic evaluation test for consistency, odor or aroma, appearance or color, taste, and overall acceptability. A one-way analysis of variance (ANOVA) was used to assess the responses in a fully randomized setup [12].

## Results and discussion

### Purification and electrophoretic analysis of *A. niger* NRC114 α-galactosidase

**Fig. 1:**
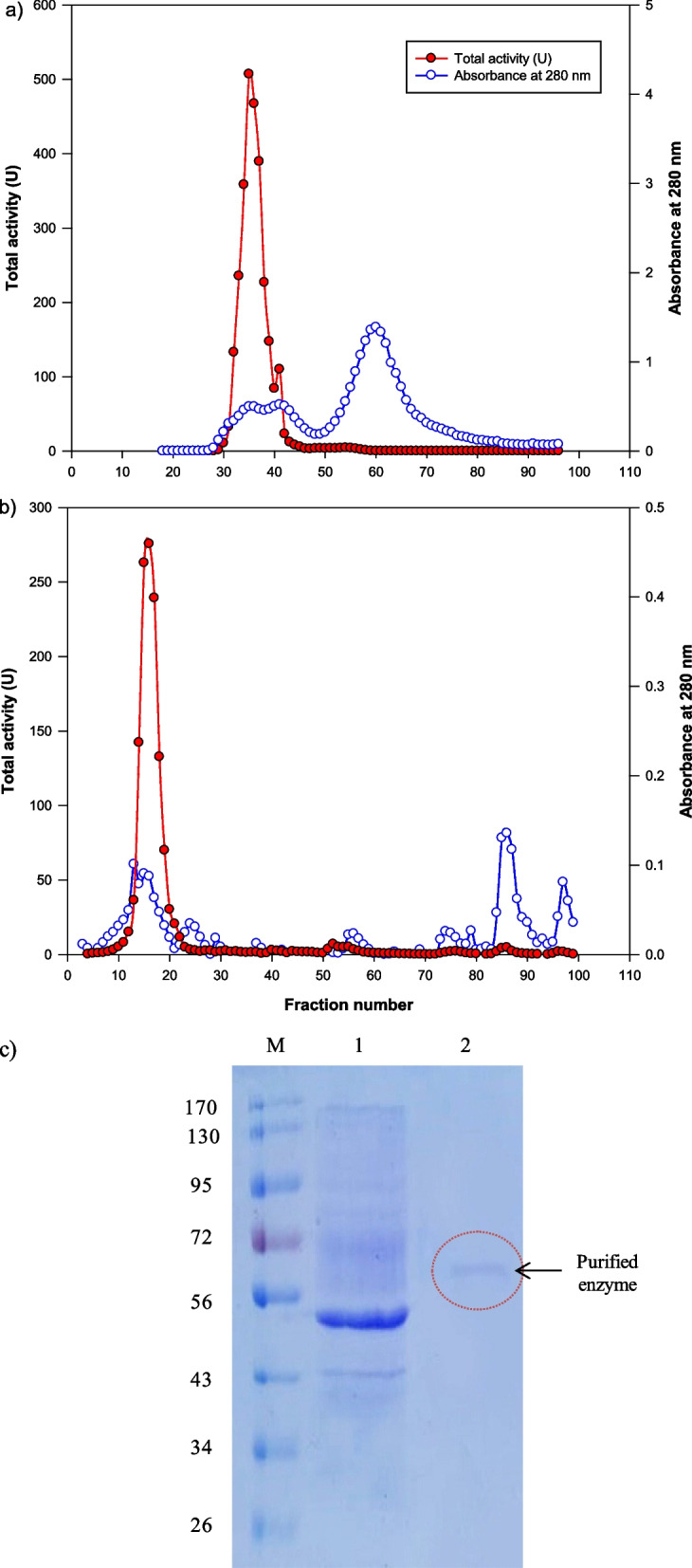
Purification of *A. niger* NRC114 α-galactosidase; (**a**) Sephadex G-100 column; (**b**) DEAE Sephadex A50 column; and (**c**) SDS-PAGE of *A. niger* NRC114 purified α-galactosidase enzyme; M: marker, lane 1: crude enzyme, and lane 2: purified α-galactosidase

The purification step depended on an optimized α-galactosidase enzyme produced by *A. niger* NRC114 through the central composite design (CCD) approach as described in our previous work [12]. Following the purification of various α-galactosidases, differential ammonium sulphate precipitations were used to isolate α-galactosidase [26]. As detailed in the Methods section, the crude enzyme was initially submitted to ammonium sulfate saturation, which revealed a total protein content of 124.40 mg and 3214.94 U of α-galactosidase activity. The activity of α-galactosidase was greater in the 60–90% saturation range among the various proteins precipitated at different ammonium sulfate saturations (Table 1). To further purify the α-galactosidase, ammounium sulfate (60–90%) fraction in a citrate phosphate buffer (pH 4.5, 0.05 M) was subjected to Sephadex G-100 column chromatography equilibrated with the same previous buffer (Fig. 1a). The step of Sephadex G-100 column chromatography resulted in a purification fold of 45 and a recovery percentage of 78.8% (Table 1). The active fractions were pooled, dialyzed against citrate phosphate buffer (pH 4.5, 0.02 M), concentrated, and subjected to ion exchange chromatography using a DEAE Sephadex A50 column. From this matrix, a stepwise gradient elution was carried out, and the fractions obtained were examined for protein and enzyme activity (Fig. 1b). The step of DEAE Sephadex A50 column chromatography resulted in a 123-fold purified enzyme. Table 1 summarizes the results of *A. niger* NRC114 α-galactosidase purification steps. When compared to the fold purification (12.7) achieved for soyabean enzyme [27] and 4.8 percent recovery for the Cicer enzyme [28], the fold purification (123-fold) and recovery (37.58%) from *A. niger* NRC114 α-galactosidase were greater.

**Table 1:** Purification of *A. niger* NRC114 *α*-galactosidase

Purification step	Total units (U)	Total protein (mg)	Specific activity (U/mg protein)	Recovery (%)	Purification fold
Crude enzyme	3780.57	698.32	5.41	100.00	1.00
Ammonium sulfate ppt. (60–90%)	3214.94	124.40	25.84	85.04	4.77
Sephadex G-100 column	2981.21	12.20	244.42	78.86	45.15
DEAE Sephadex A50 column	1420.65	2.13	666.02	37.58	123.02

The peak of activity was pooled and concentrated using Amicon centrifugal filter units (UFC9003) with a pore size of 3 kDa molecular weight cutoff (MWCO). Employing standard proteins, the molecular mass of the purified monomer enzyme that resulted from the last purification step was found to be 64 kDa using the SDS-PAGE approach (Fig. 1c). The protein that displays enzyme activity is eluted as a single peak, providing evidence that the enzyme is homogenous. According to the publications, α-galactosidases exist in several monomeric and multimeric variants with structural variety. The majority of α-galactosidases have molecular size extending between 30 and 100 kDa [29, 30]. Other α-galactosidases from *Irpex lacteus* and *Aspergillus parasiticus* also showed similar results, according to the observations [31, 32]. In this study, the purified enzyme from the DEAE Sephadex A50 column was used in all further studies.

### Reaction time, temperature, and pH effects

**Fig. 2:**
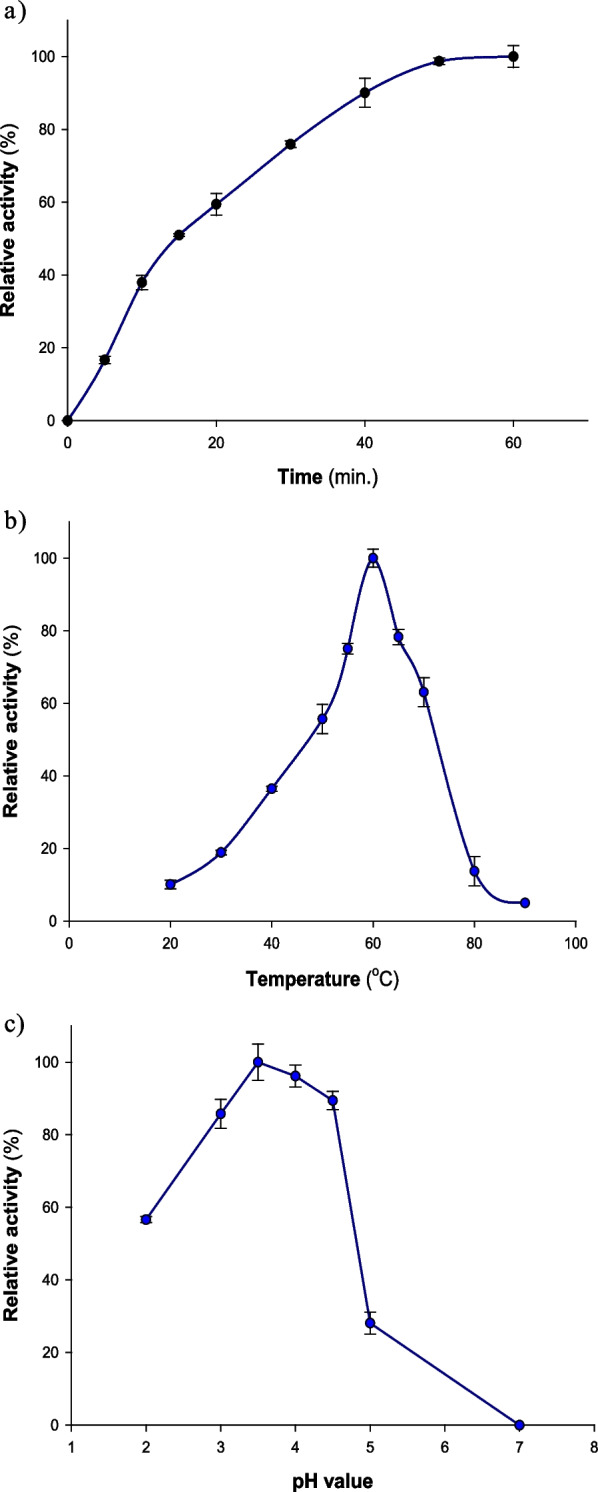
(**a**) Effect of incubation period on α-galactosidase activity. The *α*-galactosidase activity was assessed at different time intervals between (0–60 min) at 60 °C. *p*NP-α-D-Gal was incubated with the pure enzyme (25.39 U/mL) and citrate phosphate buffer pH 4.5 (0.1 M), and then the released *p*NP was determined. (**b**) Effect of temperature, and (**c**) effect of pH on α-galactosidase activity. The vertical bars show the standard deviation values, and each value reflects the mean of three replicate measurements

The α-galactosidase activity was assessed at different time intervals between (0–60 min) at 60 °C. *p*NP-α-D-Gal was incubated with the pure enzyme and citrate phosphate buffer pH 4.5 (0.1 M), and then the released *p*NP was determined. Figure 2a showed that with the increase in time, the released amount of PNP increased until the incubation time was about 50 min. After that, the enzyme activity reached its maximum extent at 60 min. This result was taken into consideration when deciding to incubate the reaction mixture for 10 min, as it was found to be suitable for the enzyme assay in the next experiments.

Figure 2b showed that the optimum temperature for α-galactosidase activity was detected at 60 °C. Before this degree, the enzyme activity exhibited a gradual increase in the range between 20 and 50 °C. On the other hand, the enzyme activity has gradually vanished at temperatures above the optimum degree, reaching 5% at 90 °C. α -galactosidases can be used directly in the processing of legume products. [33]. High-temperature processing has two major benefits: it reduces the risk of microbial contamination while also speeding up the processing time. Similar to other α-galactosidases from thermophilic fungi, including *N. fischeri* P1 [34] and* R. miehei* CAU432 [1], *A. niger* NRC114's was the most active at 60 °C, and much greater than those of the mesophilic species' α -galactosidases [35] and other α -galactosidases from seeds of Cassia, Pepino, Tachigali, Melon, and White Chickpea that have been previously reported (50 °C) [26, 29, 36]; and Cowpea α-galactosidase (35 °C) [37], and less than other α-galactosidases like *Phaseolus coccineus* α -galactosidase (70 °C) [38], *Irpex lacteus* α-galactosidase (70 °C) [31], *Thermoanaerobacterium polysaccharolyticum* α-galactosidase (77.5 °C) [39], and *Sulfolobus solfataricus* α-galactosidase (90 °C) [28].

The optimum pH value of the enzyme activity was pH 3.5, as presented in Fig. 2c. Before and after this value, the enzyme activity starts to decrease to 28% at pH 5.0. The enzyme activity completely diminished at pH 7.0, which means that the enzyme is active at acidic pH values (Fig. 2c). Most other known fungal α-galactosidases have an ideal pH in the range of pH 4.0–5.5 [40–42], whereas *A. niger* NRC114's α-galactosidase demonstrated maximal activity at a pH value of 3.5, which is more acidic.

### Thermal and pH stability

**Fig. 3:**
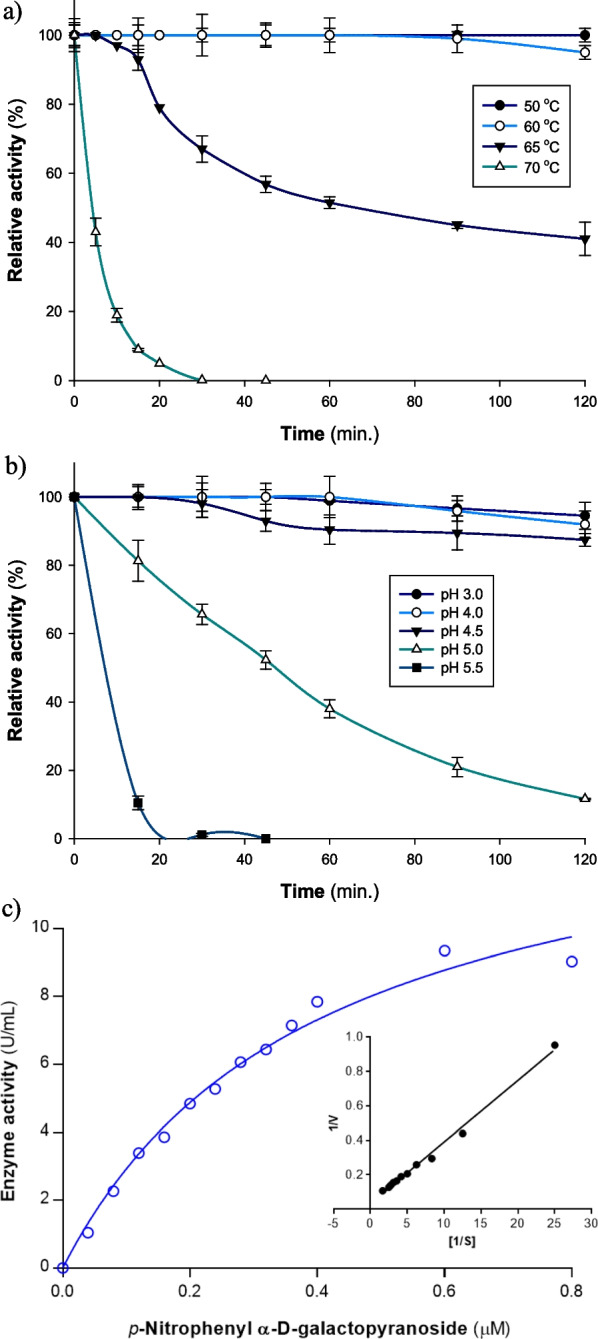
(**a**) thermal stability, and (**b**) pH stability (at 60 °C) of *A. niger* NRC114 purified *α*-galactosidase enzyme. Thermal stability was checked by incubating the enzyme (25.39 U/mL) at 50, 60, 65, and 70 °C for 120 min in a citrate phosphate buffer (50 mM, pH 3.5) and assayed periodically for its remaining activity under the standard conditions. For pH stability, the enzyme was incubated in a range of pH levels from pH 3.0 to 7.0 for 2 h at 60 °C and evaluated for its residual activity under typical circumstances. The vertical bars show the standard deviation values, and each value reflects the mean of three replicate measurements (**c**) Lineweaver–Burk plot of the reciprocal of initial velocities and *p*NP-α-D-Gal concentrations. *K*_*m*_ and *V*_*max*_ values were determined using the Lineweaver–Burk plot technique in GraphPad Prism version 6.01, and the experiments were performed in triplicate

To determine the thermal stability of *A. niger* NRC114 α-galactosidase, the enzyme was incubated at 50, 60, 65, and 70 °C for 120 min and assayed periodically for its activity under the standard conditions. *A. niger* NRC114 α-galactosidase showed complete stability at 50 °C for 120 min, and 60 °C for 90 min. With the rise in temperature, the enzyme activity started to decrease gradually with the time factor (Fig. 3a). After 10 min of incubation, *A. niger* NRC114 α-galactosidase showed complete stability at 50 and 60 °C, whereas kept 97 and 19% of its initial activity at 65 and 70 °C, respectively. After 60 min, the enzyme still showed its full stability at 50 and 60 °C, but kept half its catalytic activity after exposure to 65 °C. After 120 min of incubation, *A. niger* NRC114 α-galactosidase still showed good stability behavior and kept 100, 95, and 41% of its initial activity at 50, 60 and 65 °C, respectively (Fig. 3a). Compared to several α-galactosidases from mesophilic microbes [35], *A. niger* NRC114 exhibits significantly superior thermo-stability. It demonstrated perfect stability at 50 °C for 120 min, a period of time that is significantly longer than that of other α-galactosidases from paddy soil metagenomic ((half-life of 42 min.) [35], *Pontibacter sp*. HJ8 h. (half-life of 5 min), and *Streptomyces sp.* HJG4 (half-life of 2 min) [43]. For industrial usage, an enzyme's strong thermostability, high optimum temperature, and reasonably wide pH stability range at room temperature (30 °C) are highly valued [40].

When the purified *A. niger* NRC114 α-galactosidase was incubated in a range of pH levels from pH 3.0 to 7.0 for 2 h at 30 °C and evaluated for its residual activity under typical circumstances, the enzyme was discovered to be entirely stable in the tested pH range (data not shown). For that, the incubation temperature was increased to offer a bright stability behavior against the pH parameters. The purified *A. niger* NRC114 α-galactosidase was incubated in different pH values (pH 3.0–7.0) for 2 h at 60 °C and then assayed for its activity under standard conditions. The results obtained showed that the maximum stability was obtained at an acidic pH only, which is consistent with other previously described α-galactosidases [26, 44] proving that the α-galactosidase from *A. niger* NRC114 is acidic. After 15 min of incubation at 60 °C, *A. niger* NRC114 α-galactosidase showed varied stability and kept its complete activity at pH 3.0, 4.0, and 4.5, whereas kept 81, 10, and 3% of its initial activity at pH values of 5.0, 5.5, and 7.0, respectively. By increasing incubation time (120 min, 60 °C), the enzyme showed clear stability at acidic pH values, whereas its stability started to clearly diminish after pH 4.5. The relative percentages of remaining enzyme activity were 95, 92, 87, and 12% at pH values of 3.0, 4.0, 4.5, and 5.0, respectively, after 120 min. *A. niger* NRC114 α-galactosidase was completely inactivated at pH value of 5.5 after 30 min (Fig. 3b), which declare its acidic stability. Acidic α-galactosidases are crucial to raffinose family oligosaccharides (RFOs) metabolism [45]. *Bacillus coagulans* α-galactosidases showed stability at pH 5.0–10.0 [30] and RmgalB at pH 5.5–9.5 [1] as well as RmGal36 at pH 4.5–10.0 [46] originating from *Rhizomucor miehei* CAU432.

### Kinetic parameters

Michaelis–Menten and Lineweaver–Burk plots were used to estimate the kinetic parameters of *A. niger* NRC114 α-galactosidase with *p*NP-α-D-Gal. The *K*_*m*_ and *V*_*max*_ of *A. niger* NRC114 α-galactosidase were calculated to be 0.401 µM and 14.65 µmol min^-1^, respectively (Fig. 3c). The recorded* K*_*m*_ value on hydrolysis of  *p*NP-α-D-Gal was lower than that reported with α-galactosidase from the seeds of *Annona squamosal* (0.67 mM) [29], Cicer α-galactosidase (0.67 mM) [26], Tachigali α-galactosidase (0.45 mM) [44], Pepino α-galactosidase (0.37 mM) [47], Soya bean α-galactosidase (0.33 mM) [27]. Higher affinity for the substrate is demonstrated by *A. niger* NRC114's lower *K*_*m*_ value for the enzyme.

### Metal ions effect on α-galactosidase activity

Of the metal ions assessed; K^+^, Mg^2+^, Co^2+^, and Zn^2+^ at a concentration of 2.5 mM revealed 14.39%, 23.46%, 28.99%, and 11.87% activation on the activity of *A. niger* NRC114 purified α-galactosidase enzyme respectively; whereas Mn^2+^, Hg^2+^, and Cu^2+^ at the same concentration inhibited the enzyme activity (Table 2). This inhibition effect of Cu^2+^ and Hg^2+^ has been reported by Çelem and Önal [48] and Geng et al., [49], respectively. The enzyme was activated by 16.57% when the concentration of Zn^2+^ was increased to 10 mM. Other metals, with the exception of Mg^2+^, induced enzymatic inhibition at the same dose. Similar to α-galactosidases from *Mesorhizobium sp.* JB07, Ca^2+^ had no discernible impact on the α-galactosidase activity of *A. niger* NRC114 [50], *Streptomyces sp.* S27 [51], and *Hermetia illucens*'s gut metagenome [52]. Interestingly, EDTA had no effect on the pure α-galactosidase from *A. niger* NRC114 (Table 2), indicating that it is a non-metallo enzyme similar to the α-galactosidase found in *Annona squamosa* seeds [29].

**Table 2:** Effect of metal ions on* A. niger* NRC114 α-galactosidase activity

Metal salt	Relative activity (%)	
	2.5 mM	10 mM
Control	100.00 ± 0.00	100.00 ± 0.00
MnCl_2_	25.88 ± 0.003	43.29 ± 1.54
HgCl_2_	12.78 ± 0.06	14.68 ± 0.49
CaCl_2_	101.62 ± 1.98	ND
KCl	114.39 ± 0.00	86.68 ± 0.23
MgSO_4_	123.46 ± 3.14	101.90 ± 1.56
CoSO_4_	128.99 ± 0.67	95.07 ± 0.004
ZnSO_4_	111.87 ± 2.55	116.57 ± 2.67
CuSO_4_	93.73 ± 0.53	22.69 ± 0.37
EDTA	104.37 ± 0.88	101.55 ± 1.92

*ND* Not determined

### Assessment of different changes of soymilk yogurt with α-galactosidase treatment

#### Proximate chemical composition

The treatment of soymilk yogurt by α-galactosidase showed a minor increase (0.86%) in the moisture content of soymilk yogurt at the binning of storage time (Table 3). A gradual increase in the moisture content was detected for all samples with storage at 4 °C. During storage, all samples exhibited a reduction in total solids gradually, and the maximum reductions were found at the end of storage. The obtained results are confirmed by Kumari et al. [53], who found that plain yogurt had a lower value of total solids compared to a symbiotic type. The results showed that the total solids of the enzyme treated-soymilk yogurts were less than those of the untreated ones by 1.12% at zero time and decreased through the storage time (Table 3). The data on protein and ash changes revealed the reduction in their values during storage. The lowest values of protein were observed in the enzyme-treated yogurt at the end of storage at 4 °C (Table 3). At the beginning of storage, ash values were recorded at 0.67% and 0.74% in untreated and enzyme-treated soymilk yogurts, respectively. The fat content had been determined and reduced as the storage time increased. The highest value of fat content (4.18%) was detected with the enzyme-treated soymilk yogurt compared to untreated soymilk yogurt, which had 3.76% at zero time (Table 3). The reduction of fat content during storage may be due to the consumption of strains used in the preparation of yogurt [54].

**Table 3:** The proximate chemical composition of soymilk yogurt before and after enzyme treatment during the storage for two weeks at 4 °C

Sample	Proximate composition (g/100 g)/Storage time (days)		
	Zero	7	14
*Moisture*			
Soymilk yogurt	86.57 ± 0.15^b^	89.13 ± 0.08^b^	89.75 ± 0.07^b^
Treated soymilk yogurt	87.43 ± 0.09^a^	88.57 ± 0.04^b^	89.62 ± 0.12^b^
*Total solids*			
Soymilk yogurt	14.28 ± 0.07^b^	10.87 ± 0.08^b^	10.28 ± 0.06^b^
Treated soymilk yogurt	13.16 ± 0.03^c^	10.23 ± 0.04^b^	10.59 ± 0.3^b^
*Protein*			
Soymilk yogurt	4.79 ± 0.01^b^	4.39 ± 0.02^b^	3.85 ± 0.02^b^
Treated soymilk yogurt	3.48 ± 0.01^a^	3.26 ± 0.01^a^	3.19 ± 0.01^c^
*Fat*			
Soymilk yogurt	3.76 ± 0.02^b^	3.57 ± 0.03^a^	3.15 ± 0.09^b^
Treated soymilk yogurt	4.18 ± 0.03^a^	3.72 ± 0.01^b^	2.84 ± 0.05^a^
*Ash*			
Soymilk yogurt	0.67 ± 0.01^b^	0.52 ± 0.01^b^	0.48 ± 0.07^b^
Treated soymilk yogurt	0.74 ± 0.02^c^	0.68 ± 0.02^c^	0.55 ± 0.09^c^
*Carbohydrates*			
Soymilk yogurt	4.21 ± 0.05^b^	2.39 ± 0.02^b^	2.82 ± 0.02^b^
Treated soymilk yogurt	4.17 ± 0.04^b^	3.77 ± 0.03^c^	3.80 ± 0.03^c^
*pH*			
Soymilk yogurt	3.78 ± 0.01^b^	3.65 ± 0.01^b^	3.47 ± 0.02^b^
Treated soymilk yogurt	4.39 ± 0.02^a^	4.19 ± 0.03^a^	4.08 ± 0.06^a^
*TTA (g/L)*			
Soymilk yogurt	2.16 ± 0.02^b^	2.17 ± 0.04^b^	2.69 ± 0.03^b^
Treated soymilk yogurt	1.23 ± 0.02^a^	1.24 ± 0.03^a^	1.26 ± 0.05^a^
*Phytochemicals (Total phenols, µg/g)*			
Soymilk yogurt	52.68 ± 0.08^b^	56.97 ± 0.03^b^	60.34 ± 0.14^b^
Treated soymilk yogurt	58.73 ± 0.11^c^	61.24 ± 0.05^c^	63.67 ± 0.09^c^
*Total flavonoids (µg/g)*			
Soymilk yogurt	39.43 ± 0.07^b^	42.86 ± 0.07^b^	45.62 ± 0.10^b^
Treated soymilk yogurt	43.42 ± 0.09^c^	47.52 ± 0.09^c^	49.81 ± 0.12^c^
*Antioxidant activity*			
Ferric-Reducing power (μg of ascorbic acid equivalents/mL)			
Soymilk yogurt	124.7 ± 0.40^b^	138.4 ± 0.09^b^	142.8 ± 0.32^b^
Treated soymilk yogurt	125.2 ± 0.18^b^	141.2 ± 0.05^c^	143.9 ± 0.15^b^
ABTS assay (μg of ascorbic acid equivalents/mL)			
Soymilk yogurt	105.3 ± 0.21^b^	109.5 ± 0.17^b^	110.5 ± 0.16^b^
Treated soymilk yogurt	107.6 ± 0.17^c^	111.3 ± 0.15^c^	112.4 ± 0.18^c^

Values are expressed as Mean ± SD

Values in the same column with the same superscripts are not significantly different (*P* < 0.05).

The obtained pH values showed a gradual decrease during storage for two weeks at 4 °C in all yogurts under study. This reduction may be due to the alteration of lactose to lactic acid as well as sugar fermentation with prolonged storage time. The obtained results are confirmed by Lucey [55], who found a similar reduction in pH values in yogurt during storage. On the other hand, an increase in titratable acidity, especially in untreated enzyme yogurt, was observed compared with the treated samples. The acidity increase during storage is correlated with acid production and lactose metabolism subsequently by bacterial strains [56]. The quality of yogurt deteriorates with the increase of acidity and low pH with the increase of storage time due to bitterness and sourness.

The analysis of phytochemicals, including total phenolics and flavonoids, is given in Table 3. The increase of phytochemical content during storage was noticed, and the highest increase of phenolic content was recorded with the enzyme-treated soymilk yogurt, which was 63.67 µg/g at the end of storage in comparison with zero time (58.73 µg/g) (Table 3). A similar trend was found in total flavonoids, which increased with extended storage time. This rise may be explained by the breakdown of milk proteins caused by bacteria producing proteolytic enzymes and the release of certain phenolic acids and isoflavonoids found in soymilk [57].

The most widely used method for detecting antioxidant activity in food products is ABTS, which measures the scavenging activity of various natural products and can be used in media containing hydrophilic and lipophilic antioxidant components such as yogurt [23]. The second method used in the present study is FRAP, which is considered a powerful indicator of antioxidant status and determines the oxidative damage resulting from reactive oxygen [58]. The determination of antioxidant activity using Ferric-Reducing power and ABTS assays showed that the highest values were detected in the yogurt sample treated with α-galactosidase enzyme followed by the untreated one (Table 3). At the end of storage, a significant increase in antioxidant activity was observed compared to fresh samples in all studied samples (Table 3). In yogurt and other fermented milks, some of the antioxidant activity is related to bioactive peptides, which form during processing and fermentation, as well as during storage [59, 60]. The bioactive components in soymilk, like isoflavonoids (genistein and daidzein), vitamins, and proteins, have excellent antioxidant activity [61]. Also, the strong reducing power of prepared soymilk yogurts is attributed to the increased hydrogen ion availability as a result of fermentation by the strains used in the manufacture. The high antioxidant activity of the investigated yogurt samples correlated well with the high total phenolics and flavonoids.

### Sugar content

The degradation of oligosaccharides present in soymilk yogurt by α-galactosidase was determined by HPLC analysis. The results cited in Additional file 1: Table S1 showed a dramatic decrease in the raffinose and stachyose content of enzyme-treated soymilk yogurt. About 65% and 98% of stachyose and raffinose were eliminated after storage at 4 °C for two weeks, respectively. A dramatic decrease in lactose and fructose was observed at the end of storage, and a reversible trend was found in glucose and galactose (Additional file 1: Table S1). The increase of glucose and galactose may be correlated with the decrease of raffinose and stachyose by the enzyme hydrolytic activity.

### Sensory evaluation

The sensory evaluation for soymilk yogurts either treated or untreated for taste, color, aroma, consistency, and overall acceptability is given in Table 4. The highest scores for all attributes were recorded for plain yogurt, followed by enzyme-treated soymilk yogurt, and the least values for yogurt manufactured without enzyme treatment. A significant negative effect for storage was found on sensory properties for both soymilk yogurts and the pronounced reduction of acceptability noticed in untreated enzyme soymilk yogurt (Table 4). The consumer preferred foods with healthy, nutritional, and therapeutic properties like yogurt [62]. However, the acceptability of these foods is critical. With respect to taste, aroma, color, and overall acceptability, the yogurt prepared from soymilk after enzyme treatment indicates the panelists liked this type, which recorded scores of more than five on a ten-point scale compared to untreated soymilk yogurt. An additional processing step that may improve the low scores of treated soymilk yogurt attributes, especially sour taste or beany-aroma, is adding flavoring agents like mango or chocolate during preparation. The enhancement of undesirable tastes may be obtained by adding sugar or other natural sweeteners like allulose*.* In the study carried out by Osundahunsi et al. [63], they tried to apply orange and strawberry flavorings to soymilk yogurt and found that strawberry yogurt was preferred over the orange type.

**Table 4:** Sensory evaluation of plain and soymilk yogurts

Attributes	Plain yogurt	Untreated soymilk yogurt	Treated-soymilk yogurt
Taste	9.58 ± 0.23^a^	4.56 ± 0.08^b^	8.74 ± 0.15^c^
Colour	9.92 ± 0.18^a^	7.83 ± 0.15^b^	9.23 ± 0.17^a^
Aroma	8.95 ± 0.15^a^	6.29 ± 0.16^b^	7.82 ± 0.13^a^
Consistency	8.16 ± 0.19^a^	5.34 ± 0.12^b^	7.95 ± 0.12^a^
OAA	9.13 ± 0.24^a^	5.26 ± 0.17^b^	8.67 ± 0.09^c^

Values in the same row with the same superscripts are not significantly different (*P* < 0.05)

*OAA* Overall acceptability

### Volatile compounds

The extraction of volatile compounds using SPME and analysis by GC–MS showed that a total of 36 volatile compounds had been identified and are given in Additional file 1: Table S2. These identified volatile compounds belong to various chemical groups and can be classified as follows: 11 ketones as well as aldehydes, 7 esters, 6 acids, 5 alcohols, 3 terpenes, and 4 sulfur-containing compounds. Many of these volatile compounds are formed after fermentation and subsequent chemical changes during storage [64]. Acetaldehyde is the most important volatile compound that has a fresh, fruity, pungent taste, and it does originate from the metabolism of lactose, the decarboxylation of pyruvate, or the intermediate acetyl coenzyme A [65]. In all studied yogurt samples, acetaldehyde was found with significant variation either at zero time or after storage (Additional file 1: Table S2). The alcohol dehydrogenase enzyme had the ability to metabolize acetaldehyde into ethanol [66]. Also, the reduction of acetaldehyde during storage correlated well with the evaporation and/or hydrolysis by microbial enzymes.

It is well known that acetaldehyde can be oxidized easily, and the formation of acetate has occurred. The obtained results are confirmed by the study carried out by Hussein et al. [67] on plain and fortified yogurts with various additives. Among the identified aldehydes, hexanal, which gives a fruity note and originates from β-oxidation, was found with concentrations of 1.15% and 2.82% in untreated and treated soymilk-yogurt, respectively (Additional file 1: Table S2**)**. The obtained data are in good agreement with previous studies carried out by Saint-Eve et al. [68] and Donkor et al. [69]. Another major volatile compound in yogurt is acetoin (diacetyl), which has a buttery, fatty, pungent odor and is formed by the acetolactate decarboxylase enzyme. The importance of acetoin in yogurt flavor appears when a low concentration of acetaldehyde has occurred [70]. Table S2 showed a reduction in acetoin in all yogurt samples at the end of storage. This phenomenon may be due to microbial hydrolysis by enzymes and the formation of new volatile compounds [71]. During storage in the refrigerator, acetoin was recorded in all studied yogurt samples, with the highest concentration in treated soymilk yogurt (1.70%) and the least concentration (1.01%) recorded in untreated soymilk yogurt (Additional file 1: Table S2). The previous studies by Chammas et al. [72] and Condurso et al. [64] confirmed our data.

Another volatile compound class that is formed via β-oxidation is ketones**.** The ketones such as 2-nonanone, 2-heptanone, and 2-propanone are responsible for the metallic flavor in ultrahigh-temperature (UHT) milk. The high concentrations of ketones such as 2,3-pentanedione and 2-heptanone, which are characterized by creamy and buttery and creamy and fresh flavors, respectively, impact the flavor in yogurt [73]. 2,3-Pentanedione is formed during the metabolism of isoleucine by α-aceto-α-hydroxybutyrate [74]. The most significant ketones were recorded as 2-heptanone, 2-nonanone, and 2-undecanone (Additional file 1: Table S2). Both soy yogurts prepared from either untreated or enzyme-treated soymilk exhibited higher concentrations of 2-undecanone, 2-nonanone (creamy and fresh notes), and 2-heptanone with a significant reduction at the end of storage (Additional file 1: Table S2). Herein, we could identify six ketones during the storage of yogurt samples. Among the identified ketones, 2,3-pentanedione and 2-nonanone were recorded in all samples, while 2,3-butanedione, 2-heptanone, and 2-butanone were identified in soymilk yogurts (Additional file 1: Table S2). This variation may be due to the chemical composition of milk and the effect of thermal treatment [70]. The identified ketones in the present study were found in small concentrations in all yogurt samples. The increase in ethanol in the current study is confirmed by several researchers [75]. On the other hand, our results contrast with those of Vah and Hruškar [76] and Ekinci and Gurel [71], who reported a significant reduction in ethanol during storage. With the increase of ethanol during storage, a reduction of acetaldehyde has been observed depending on the type of yogurt. Our results are in agreement with Demirci et al. [76].

In the current study, a total of seven esters were identified, and their concentrations are given in Additional file 1: Table S2. Ethyl acetate, which is responsible for the fruity and pineapple flavors, was recorded. The highest concentration of ethyl acetate was found in enzyme soymilk yogurt (2.85%), whereas the lowest concentration was recorded at 0.25% in an untreated soymilk yogurt sample at zero time (Additional file 1: Table S2). The presence of ethyl acetate has been recorded in yogurt samples [68]. Esters such as ethyl acetate and ethyl hexanoate play an important role in yogurt flavor and are responsible for the reduction of unpleasant odor and short-chain fatty acids [77]. Additional file 1: Table S2 showed a total of six volatile acids, including acetic, butanoic, pentanoic, hexanoic (capric acid), octanoic, and decanoic acids, which were identified with significant concentrations in soymilk yogurts. During storage, the soymilk yogurts had a higher concentration of the aforementioned acids, which may explain the sour taste and low scores of sensory attributes (Table 4). The concentrations of acetic acid were 11.91% and 7.33% in the untreated soymilk yogurt and the enzyme-treated one, respectively, at the end of storage. The study of Terpou et al. [78] found similar data for probiotics in yogurt after enrichment with bran from wheat. The analysis of the studied yogurt samples revealed four sulfur-containing compounds that had been identified, as shown in Table S2. Dimethyl sulfide was the major sulfur compound in enzyme soymilk yogurt with a concentration of 7.42%, followed by 3-(methylthio)-1-propanol, which recorded 4.80% at zero time, and these compounds were significantly reduced at the end of storage.

## Conclusions

Purification of *A. niger* NRC114 α-galactosidase yielded a 123-fold purified enzyme. The characterization of the pure enzyme revealed that the pure enzyme has high levels of pH and thermal stability, especially at acidic conditions. The relative lower *K*_*m*_ value of the enzyme under study indicates its higher affinity for substrate, which is highly recommended. The treated soymilk yogurt showed an increase in total phenolic and flavonoid contents, as well as several detected volatile compounds. In addition, the enzymatic action in the hydrolysis of raffinose family oligosaccharides was confirmed. These findings suggest that this enzyme has the potential to be used in a variety of important future studies, including the treatment of Fabry's disease, blood group conversion, and the eradication of immunogenic α-gal epitopes in xenotransplantation.

## Supplementary Information

**Additional file 1: Table S1: **HPLC analysis of sugars (%) in soymilk yogurts. **Table S2:** Effect of storage on volatile compounds of soymilk yogurt samples

## Data Availability

This article has all the data that was created or evaluated during this investigation.
