# Supplementary material for: Biochemical characterization and insights into the potency of the acidic Aspergillus niger NRC114 purified α-galactosidase in removing raffinose family oligosaccharides from soymilk yogurt
Source: BMC Biotechnol. 2023 Jan 31;23:3. doi: 10.1186/s12896-023-00773-x (PMC9887927; doi:10.1186/s12896-023-00773-x)
Supplement: Supplementary file 1 — Additional file 1: Table S1: HPLC analysis of sugars (%) in soymilk yogurts. Table S2: Effect of storage on volatile compounds of soymilk yogurt samples [file 12896_2023_773_MOESM1_ESM.docx]

Table S1: HPLC analysis of sugars (%) in soymilk yogurts

| Sugars | Treatment |  |
| --- | --- | --- |
|  | Untreated soymilk yogurt | Treated soymilk yogurt |
| Lactose |  |  |
| Zero time | n.d | 0.03 |
| 14 days | 1.26 | 0.01 |
| Galactose |  |  |
| Zero time | 0.54 | 0.07 |
| 14 days | 1.73 | 2.45 |
| Glucose |  |  |
| Zero time | 0.01 | 0.89 |
| 14 days | 0.67 | 1.37 |
| Fructose |  |  |
| Zero time | 0.62 | 3.15 |
| 14 days | 0.03 | 0.95 |
| Sucrose |  |  |
| Zero time | 1.34 | 2.83 |
| 14 days | 0.85 | 1.97 |
| Raﬃnose |  |  |
| Zero time | 0.52 | 0.31 |
| 14 days | 0.49 | 0.18 |
| Stachyose |  |  |
| Zero time | 0.98 | 0.05 |
| 14 days | 0.62 | 0.02 |

n.d : not detected

Table S2: Effect of storage on volatile compounds of soymilk yogurt samples

| Volatile compounds | LRI^a^ | Zero time |  | 14 days storage | |
| --- | --- | --- | --- | --- | --- |
|  |  | Untreated | Enzyme-treated soymilk yogurt | Untreated | Enzyme-treated soymilk yogurt |
| Aldehydes and ketones |  |  |  |  |  |
| Acetaldehyde | 709 | 20.46 | 25.23 | 19.18 | 23.45 |
| 2-Propanone | 772 | 1.34 | 1.23 | 2.46 | 1.52 |
| 2-Butanone | 865 | 1.37 | 2.74 | 1.13 | 0.94 |
| Butanal | 879 | 0.83 | 1.80 | 0.92 | 3.87 |
| 2,3-Butanedione | 954 | 2.78 | 0.62 | 0.79 | 3.32 |
| 2,3-Pentanedione | 1043 | 0.87 | 0.69 | 0.54 | 2.24 |
| Hexanal | 1069 | 1.15 | 2.82 | 7.35 | 0.69 |
| 2-Heptanone | 1174 | 1.32 | 1.67 | 1.38 | 0.61 |
| Acetoin | 1287 | 1.01 | 1.70 | 0.67 | 0.95 |
| 2-Nonanone | 1381 | 0.41 | 1.01 | 0.54 | 1.22 |
| Nonanal | 1385 | 0.36 | 2.19 | 0.41 | 2.80 |
| Esters |  |  |  |  |  |
| Ethyl acetate | 853 | 0.25 | 2.85 | 1.09 | 0.81 |
| Methyl propanoate | 874 | 0.34 | 0.59 | 0.93 | 0.91 |
| Methyl butyrate | 963 | 0.83 | 1.90 | 0.50 | 2.70 |
| Ethyl butyrate | 1022 | 0.32 | 1.64 | 0.02 | 0.97 |
| Isopropyl pentanoate | 1075 | 1.38 | 1.49 | 0.01 | 0.54 |
| Butyl butanoate | 1204 | 0.34 | 1.27 | 0.35 | 0.68 |
| Ethyl hexanoate | 1225 | 1.89 | 0.89 | 0.33 | 1.40 |
| Acids |  |  |  |  |  |
| Acetic acid | 1443 | 1.02 | 1.60 | 11.91 | 7.33 |
| Butanoic acid | 1612 | 2.25 | 2.34 | 1.51 | 1.40 |
| Pentanoic acid | 1723 | 2.18 | 0.65 | 0.71 | 1.24 |
| Hexanoic acid | 1832 | 10.61 | 0.62 | 0.44 | 0.7 |
| Octanoic acid | 2043 | 2.56 | 0.52 | 0.52 | 0.83 |
| Decanoic acid | 2236 | 0.46 | 1.78 | 0.85 | 2.62 |
| Alcohols |  |  |  |  |  |
| Ethanol | 905 | 10.31 | 12.21 | 29.85 | 19.62 |
| 1-Hexanol | 1342 | 0.28 | 0.79 | 0.67 | 0.61 |
| 2-Ethylhexanol | 1475 | 6.05 | 2.25 | 0.58 | 1.29 |
| 1-Octanol | 1543 | 1.57 | 1.70 | 0.48 | 0.79 |
| 1-Nonanol | 1648 | 0.09 | 1.62 | 0.46 | 2.31 |
| Terpenes |  |  |  |  | 2.34 |
| β-Myrcene | 1142 | 2.94 | 1.170 | 1.31 | n.d |
| d-Limonene | 1185 | 1.85 | 1.95 | 0.5 | 2.94 |
| Terpinen-4-ol | 1604 | 0.43 | 2.16 | 1.12 | 1.05 |
| Sulfur-containing compounds | | | | | |
| Dimethyl sulfide | 1086 | 4.56 | 7.42 | 3.67 | 1.16 |
| 4-Methylthiazole | 1623 | 3.28 | 0.62 | 2.49 | 1.56 |
| 2-Acetylthiazole | 1638 | 2.94 | 2.76 | 0.82 | 1.48 |
| 3-(Methylthio)-1-propanol | 1705 | 7.82 | 4.80 | 2.56 | 0.91 |

^a^ Linear retention index; Values are expressed as relative area percentages; n.d: not detected
